# A simple strategy to decrease fatal carotid blowout syndrome after stereotactic body reirradiaton for recurrent head and neck cancers

**DOI:** 10.1186/1748-717X-8-242

**Published:** 2013-10-18

**Authors:** Gozde Yazici, Tolga Yusuf Sanlı, Mustafa Cengiz, Deniz Yuce, Melis Gultekin, Pervin Hurmuz, Ferah Yıldız, Faruk Zorlu, Fadil Akyol, Murat Gurkaynak, Gokhan Ozyigit

**Affiliations:** 1 Hacettepe University Faculty of Medicine, Department of Radiation Oncology, Sihhiye, Ankara, Turkey

**Keywords:** Stereotactic radiotherapy, Reirradiation, Head and neck cancer, CyberKnife, Fractionation

## Abstract

**Background:**

This study aimed to compare the therapeutic outcomes and fatal carotid blow out syndrome (CBOS) incidence rates between two different stereotactic body radiotherapy (SBRT) protocols.

**Methods:**

The study included 75 patients with inoperable locally recurrent head and neck cancer treated with SBRT in our department between June 2007 and March 2011. The first 43 patients were treated sequentially (group I). Then our SBRT protocol was changed due to the high rate of CBOS, and the following 32 patients were treated every other day in a prospective institutional protocol (group II).

**Results:**

Median overall survival in group I and group II was 11 months and 23 months, respectively (P = 0.006). We observed 11 cases of CBOS. Only 1 of 7 patients (14%) with CBOS survived in group I, whereas 2 of 4 patients (50%) in group II remain alive. CBOS free median overall survivals were 9 months, and 23 months in group I and group II respectively (P = 0.002). The median radiation dose received by the carotid artery in patients with CBOS was 36.5 Gy (range: 34–42.8 Gy), versus 34.7 Gy (range: 0–44 Gy) in the patients that didn’t have CBOS (P = 0.15). CBOS did not occur in any of the patients with a maximum carotid artery radiation dose <34 Gy.

**Conclusions:**

Every other day SBRT protocol for re-irradiation of recurrent head and neck cancer is promising in terms of decreasing the incidence of fatal CBOS.

## Background

The rate of local recurrence or persistent disease in patients with locally advanced head and neck cancer is 3%-50%, despite the use of multidisciplinary treatment modalities [1-3]. Treatment choices for such patients are sparse and outcomes are not satisfactory. The prognosis is reported to be better in patients undergoing surgery, which can be performed in only a minority of patients [4-6]. Even when surgery is performed additional local treatment is necessary because of the high rate of local recurrence [4-8].

Re-irradiation is associated with an increase in local control and overall survival. Three-dimensional conformal radiotherapy (3D-CRT) and intensity-modulated radiotherapy (IMRT) series have reported local control rates of 60%-70% for re-irradiation in patients with recurrent head and neck cancer; however, the occurrence of serious toxicity was high (10%-40%) [9-11]. Stereotactic body radiotherapy (SBRT) is a relatively new technique for re-irradiation of recurrent head and neck cancer. SBRT facilitates administration of high doses of radiation to the tumor while offering maximal protection to adjacent organs. We recently reported the results of SBRT in this group of patients [12]. Although our local control rate was higher than that in IMRT series, the occurrence of carotid blowout syndrome (CBOS) was relatively high, and as such we subsequently altered our SBRT protocol. The present study aimed to compare the local control rate, overall survival rate, and toxicity rate of the 2 SBRT protocols (our previously reported and newer protocols) for re-irradiation in patients with locally recurrent head and neck cancer. Furthermore, we particularly assessed whether fatal CBOS incidence changed after every other day SBRT schedule.

## Methods

The study included 75 patients with inoperable recurrent head and neck cancer that were treated with SBRT in our department between June 2007 and March 2011. The first 43 patients were treated sequentially (group I), and then our SBRT protocol was changed due to the high rate of CBOS, so the following 32 patients were treated every other day in a prospective institutional protocol (group II). The study protocol was approved by the Hacettepe University Faculty of Medicine Institutional Review Board, and written informed consent was obtained from all the patients before undergoing re-irradiation. Distant metastasis was ruled out based on bone scintigraphy, thoracic computed tomography (CT), abdominal CT, or positron emission tomography (PET) findings. All local instances of recurrence were confirmed either radiologically or histopathologically.

Patients were immobilized using a head and neck thermoplastic mask. CT and magnetic resonance imaging (MRI) (slice thickness: 1 mm) were performed with the patients in the treatment position. The images obtained were then fused for contouring. Gross tumor volume (GTV) was delineated as the planning target volume (PTV). Multiplan (Accuray Inc., Sunnyvale, CA) software was used for inverse planning. The treatments were delivered via a CyberKnife (Accuray Inc., Sunnyvale, CA).

We previously reported that there was a significant risk of CBOS in patients whose carotid arteries were surrounded by the tumor >270° circumferentially [12]; therefore, we used CT angiography to follow-up this particular subgroup of patients. All patients were informed briefly about the importance of nasal bleeding and were told to contact us immediately if they experienced any bleeding-even minor bleeding. Patients were followed-up monthly for the first 3 months post SBRT, and then every 3 months for 2 years.

Overall survival, progression free survival, and survival curves were computed using the Kaplan-Meier method and compared by Cox-Mantel log-rank test. All statistical analysis was performed using the SPSS 15.0 software.

## Results

**Figure 1:**
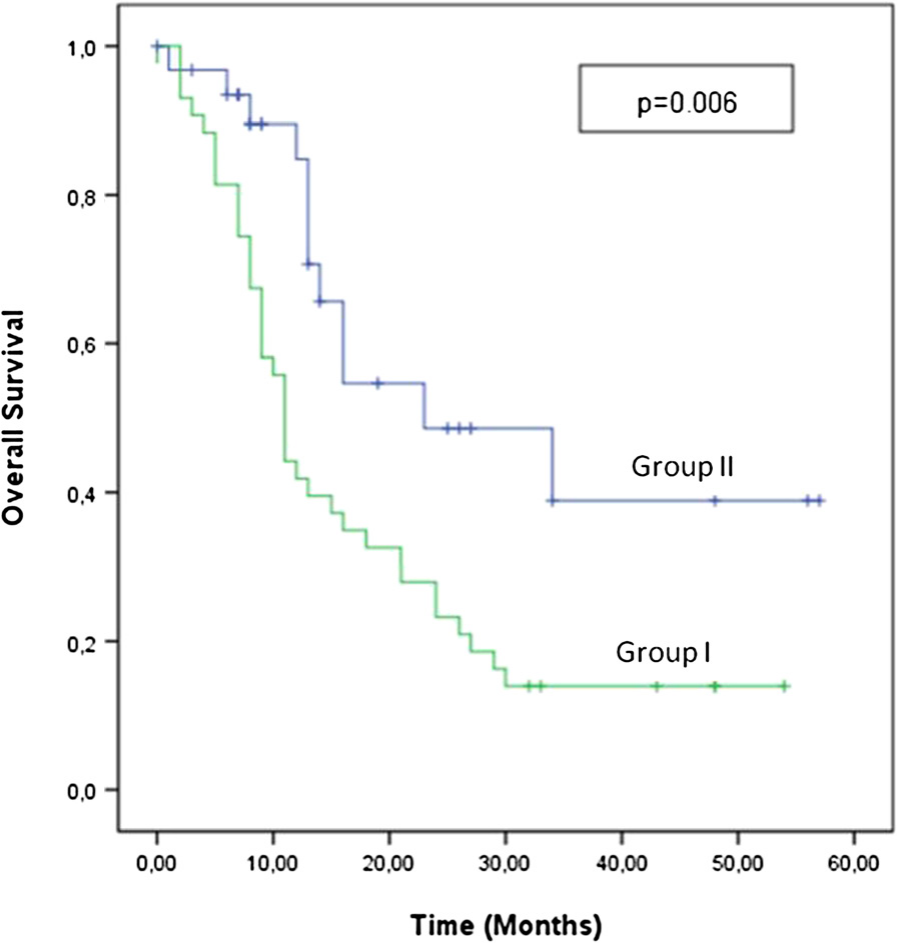
Overall survival analysis.

Patient characteristics are summarized in Table 1. Median age of all the patients was 53 years (range: 15–87 years). There wasn’t a significant difference in demographic or clinical characteristics between the 2 groups. The most common site of re-irradiation was the nasopharynx (n = 34 patients), followed by the oral cavity and larynx. The SBRT treatment characteristics are given in Table 2. Median overall survival was 14 months (range: 10.8-17.2 months) for all patients. Median overall survival in group I and group II was 11 months and 23 months, respectively (P = 0.006) (Figure 1). The overall survival rate in group I and group II was 42% and 84% at 12 months, and 23% and 38% at 24 months, respectively. Median CBOS free overall survival was 9 months (range: 6.5-11 months) in group I, and was 23 months (range: 10.8-38.1 months) in group II (P = 0.002).

**Table 1:** Patient characteristics

	**Group 1**	**Group 2**	**P**
**Sex (n)**			
-Female	22	9	0.03
-Male	21	23	
**Age at SBRT (years)**			
-Minimum and maximum	19-87	15-83	>0.05
-Median	53	57	
**SBRT site (n)**			
-Nasopharynx	19	15	>0.05
-Oral cavity	8	4	
-Paranasal	8	3	
-Larynx	4	8	
-Hypopharynx	4	2	
**Dose of primary radiation (Gy)**			
-Minimum and maximum	30-77.4	38-78	>0.05
-Median	65	64	
**Time between primary radiation treatment and SBRT (months)**			
-Minimum and maximum	9-232	10-300	>0.05
-Median	35	36	

**Table 2:** Treatment characteristics

**Treatment characteristics**	**Group 1**	**Group 2**	**P**
**SBRT dose (Gy)**			
-Range	25-35	15-35	
-Median	30	30	
**Target volume (mL)**			
-Range	4-214	5-166	0.551
-Median	54	46	
**Number of fractions**			
-Range	3-5	4-6	
-Median	5	5	
**Carotid artery dose (Gy)**			
-Range	2.5-42.5	6.8-39.1	>0.05
-Median	35	34.9	
**Temporomandibular joint dose (Gy)**			
- Range	0-42	0-33.5	0.217
-Median	24	14.7	
**Temporal lobe dose (Gy)**			
-Range	0-44.1	0-35.9	
-Median	21.7	21.5	
**Conformity index**			
-Range	1.1-2.46	1.14-2.23	
-Median	1.57	1.54	
**Homogeneity index**			
-Range	1.14-1.67	1.15-1.43	
-Median	1.32	1.28	
**Prescribed isodose (Gy)**			
-Range	60-88	70-87	
-Median	77	78	
**Collimator size (mm)**			
- Range	10-40	12.5-35	
-Median	20	20	
**Beam number**			
-Range	142-369	121-481	
-Median	206	200	

**Figure 2:**
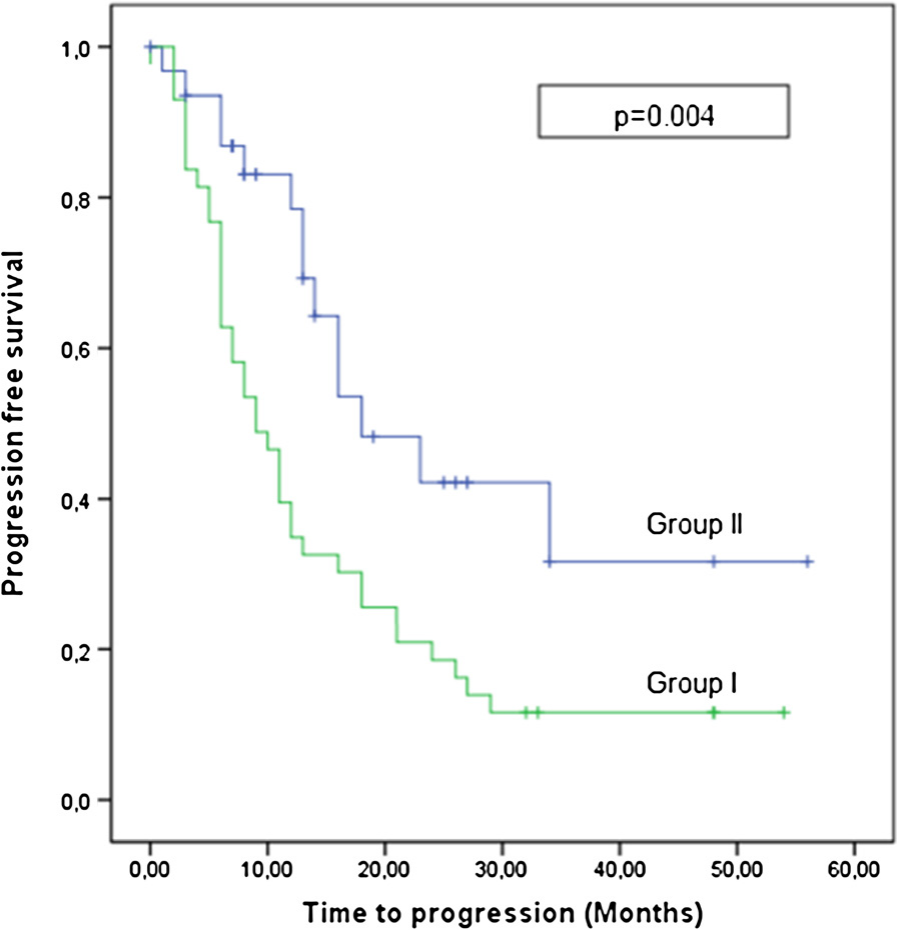
Progression-free survival.

Complete response was achieved in 12 patients (16%), 17 patients (22.7%) had partial response, and stable disease was observed in 29 patients (38.7%). Ultimate local control was achieved in 77.4% of the patients. The local control rate was 67.5% in group I, versus 90.6% in group II (P = 0.029). Progression-free survival was 13 months for all patients, 9 months in group I, and 18 months in group II (P = 0.004) (Figure 2). Tumor size was considered to be possible prognosticator. However, we could not detect any significant difference in time to progression, overall survival, or risk of bleeding between the patients with GTV ≤52 versus GTV >52 mL.

The most common grade II and higher toxicities were dysphagia (16%) and CBOS (14.7%). Dysphagia was observed in 10 patients (23%) in group I, versus 3 patients (6.3%) in group II (P = 0.047). In all, 7 patients (16%) in group I had CBOS, but only 1 of them survived, whereas 4 patients (12.5%) in group II had CBOS and to date 2 remain alive. The median radiation dose received by the carotid artery in patients with CBOS was 36.5 Gy (range: 34–42.8 Gy), versus 34.7 Gy (range: 0–44 Gy) in the patients that didn’t have CBOS (P = 0.15). CBOS did not occur in any of the patients with a maximum carotid artery radiation dose <34 Gy. The treatment characteristics of patients with CBOS are given in Table 3. The biologic effective dose (BED) for late responding tissues assuming an α/β ratio of 3 were calculated for the SBRT scheme and for the cumulative dose received by the patient. The median BED of SBRT were 95 Gy_3_, and 92 Gy_3_ for patients with and without CBOS respectively (p = 0.8). The cumulative BED was 203 Gy_3_ in patients with CBOS whereas it was 198 Gy_3_ in patients without CBOS (p = 0.7).

**Table 3:** Clinical and treatment characteristics of patients with carotid blow-out syndrome

**Patient number**	**SBRT scheme**	**GTV volume (ml)**	**SBRT site**	**Dose of primary radiotherapy (Gy)**	**SBRT dose (Gy)/fx number**	**Maximum carotid artery point dose (Gy)**	**Local control**
1	I	157	Hypopharynx	60	30/5	41.8	Complete response
2	I	173	Retromolar trigone	60	35/5	59	Progression
3	I	130	Larynx	60	30/5	39.4	Complete response
4	I	29	Nasopharynx	70	35/5	46.6	Partial response
5	I	101	Nasopharynx	66	30/5	38.5	Complete response
6	I	37	Nasopharynx	65	30/5	37.5	Progression
7	I	50	Nasopharynx	66	32/5	40.7	Stabile
8	II	55	Nasopharynx	66	32/6	46	Partial response
9	II	127	Larynx	70	30/5	37.5	Stabile
10	II	30	Hypopharynx	64	30/5	41	Stabile
11	II	51	Oral cavity	70	30/5	38.5	Stabile

GTV: gross tumor volume, Gy: gray, fx: fraction, SBRT: strereotactic body radiotherapy, I: consecutive day, II: every other day.

The circumference of the carotid artery entrapped by the tumor was calculated in each patient; median carotid artery wall entrapment by the tumor in all patients was 180°, versus 270° in the patients that had bleeding were considered. CBOS was not observed in patients with lesions entrapping <180° of the carotid artery.

## Discussion

Current study compared 2 SBRT protocols for re-irradiation in locally recurrent head and neck cancer patients in terms of therapeutic outcomes and toxicity. We demonstrated that a simple strategy change in fractionation dramatically increase survival and decrease fatal CBOS and other serious SBRT related toxicities. The 2-year overall survival rate in every other day protocol is among the best to be reported in the literature.

With conformal radiotherapy or IMRT modalities radiation doses of 60 Gy resulted in response rates of 60%-70% in patients with recurrent head and neck cancer; however, the occurrence of grade 3 or higher serious late effects was also high (40%) [10,11,13]. There is a growing body of evidence for re-irradiation of head and neck cancer patients with SBRT protocols. George et al. reported a 26% local control rate and 22% overall survival rate without grade 4 or higher side effects in response to doses of 20–30 Gy administered in 5 fractions [14]. Another study that included 36 patients with recurrent head and neck cancer that were re-irradiated with a median dose of 30 Gy in 3–5 fractions reported that the 1-year local control rate was 61%, but that grade 3 or higher side effects occurred in 37% of patients^3^. In an earlier study we compared 3D-CRT and SBRT in 51 recurrent nasopharyngeal cancer patients; the 2-year local control rate was similar in both groups (80% versus 82%), but serious late effects were significantly less common in the SBRT group (48% versus 21%, P < 0.05) [15]. Furthermore we noticed that fatal CBOS rates were much higher than the previously reported reirradiation series [16-18]. Since we used very high dose per fraction in a previously irradiated patient the interval between two consecutive fractions might be inadequate in terms of sublethal damage repair in critical tissues. In the light of this hypothetical idea, which is also supported by the study of King et al. as they showed that every other day protocol resulted in a reduced rectal toxicity rate in prostate cancer patients treated with SBRT, we decided to change our SBRT fractionation schedule by increasing the interval between fractions [19]. Consequently we observed that group II fared better than sequential group in terms of toxicities, particularly in terms of fatal CBOS. In a recent article Yamazaki et al. concluded that the every other day treatment may have a potential impact on adverse toxicities as most papers reporting CBOS used SBRT in consequtive days [20]. Our results supports the idea by Yamazaki et al., but in contrast to the literature we observed CBOS also in every other day treatment group [21,22].

The current BED formula has not been validated for the large doses per fraction however it is used to evaluate different schemas in terms of adverse reactions. We calculated the BED for late responding tissues in order to reach a cut off value for the CBOS. However, we couldn’t find a specific cut-off point for the carotid artery cumulative dose.

It may be argued that the median follow up time for every other day protocol was inadequate to asses CBOS. However, it is noteworthy that all cases of CBOS were observed in the first year of SBRT in group I, and minimum follow up was longer than 1 year with a median follow up of 23 months in group II. It seems that the decrease in fatal CBOS incidince resulted in an increase in OS rates of patients in group II.

We previously reported that tumor surrounding >180° of the carotid artery are associated with a significantly elevated risk of bleeding in stereotactic re-irradiation of recurrent head and neck cancer. In current study we performed further analysis on the relationship between the carotid artery radiation dose and the risk of bleeding. We noticed that all patients with bleeding had a median carotid artery radiation dose above 34 Gy. Likewise, none of the patients with a tumor surrounding <180° of the carotid artery experienced bleeding. Therefore clinicians should be aware of dose limits of carotid arteries to prevent that fatal complication.

## Conclusion

In the absence of a prospective randomized trial, our current institutional SBRT protocol for re-irradiation of locally recurrent head and neck cancer is to treat patients every other day. If an individual SBRT plan violates the limits of the carotid artery dose mentioned above or the tumor entraps the carotid artery more than 180°, we recommend IMRT with conventional fractionation. However, further prospective trials are required to clarify both the role of SBRT and the optimal fractionation regimen in locally recurrent head and neck cancers.

## Competing interests

The authors report no actual or potential conflicts of interest relevant to the material presented herein.

## Authors’ contributions

GO is the principal investigator, planned the design of the study and helped to draft the manuscript. GY drafted the manuscript, participated in the design, performed the statistical analysis. MC have made substantial contributions to conception and design. DY participated in analysis and interpretation of data. MG, PH, FY, FZ, FA, MG revised the manuscript critically for important intellectual content. All authors read and approved the final manuscript.
